# Multimodal method for landslide risk analysis

**DOI:** 10.1016/j.mex.2019.04.012

**Published:** 2019-04-16

**Authors:** William Pollock, Alex Grant, Joseph Wartman, Grace Abou-Jaoude

**Affiliations:** a Dept. of Civil and Environmental Engineering, University of Washington, 201 More Hall, Seattle, WA 98195, United States; b Dept. of Civil Engineering, Lebanese American University, 304 Bassil Bldg., Byblos, Lebanon

**Keywords:** Multimodal Method for Landslide Risk Analysis, Mass-wasting, Hazard, Coseismic, Precipitation, Modes of failure

## Abstract

Quantitative landslide risk analysis is a key step in creating appropriate land use policies. However, regional scale landslide hazard and risk studies are traditionally based on a single, infinite-slope style of failure, belying the differing consequences of a diverse range of failure modes. In this paper we expand an existing multimodal coseismic landslide hazard model to create a method for multimodal, multi-trigger quantitative landslide risk analysis and apply it to the country of Lebanon.

• Physics-based, mode-specific models for coseismic and precipitation-induced landslides capture the effects of multiple failure types and triggering scenarios.
• A new model for analyzing slope stability against rotational failures allows for efficient, regional scale assessments.
• Open-source mapping of built-up area is used to identify elements at risk.

## Graphical abstract

**Figure:**
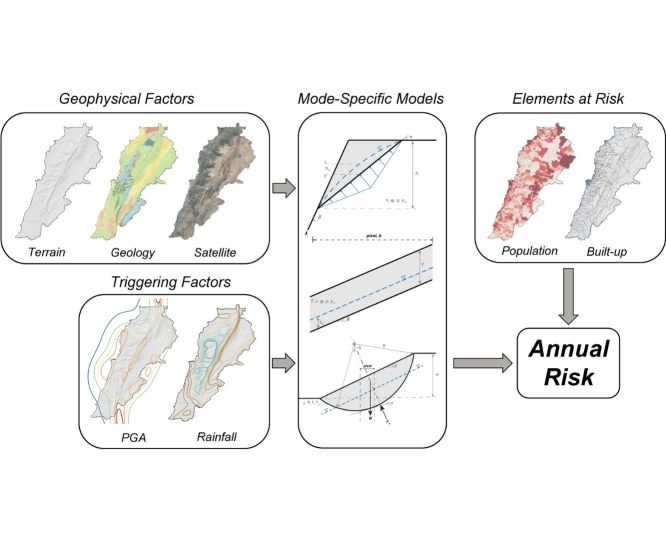

**Specifications Table**

**Table:** 

Subject Area:	Engineering
More specific subject area:	Landslide Risk
Method name:	Multimodal Method for Landslide Risk Analysis
Name and reference of original method:	Multimodal Method for Coseismic Landslide Hazard Assessment Grant, A., Wartman, J., Abou-Jaoude, G. (2016). Multimodal method for coseismic landslide hazard assessment. *Eng. Geol.*, 212, 146-160, https://doi.org/10.1016/j.enggeo.2016.08.005
Resource availability:	NA

## Quantitative landslide risk model description

### Modes of failure

Traditional large-scale landslide hazard and risk analyses rely on the simplistic infinite slope method to capture all modes of mass wasting. However, unique modes of mass wasting cause unique consequences to the human environment. Slow moving landslides tend to impact static infrastructure, especially extensive transportation networks, while rapid, localized flows pose greater risk to human lives. General infinite slope assumptions miss nuanced consequences of unique modes of movement. Hungr et al. [1] revise the traditional Varnes classification of landslide types to describe 32 unique modes of sliding. After Grant et al. [2], we simplify this classification to three modes of movement—1) shallow, planar soil slides; 2) rockfalls; and 3) rotational, coherent slumps in soil and rock. We further differentiate between triggering factors: seismic shaking which produces short-runout, disrupted failures and rainfall which can lead to confined, long runout debris flows. Statistical studies have found a correlation between slope and landslide mode (e.g [3,4]). We divide the terrain into slope-based zones susceptible to each failure mode based on previous work and existing landslide inventories in Lebanon [2,5]. Slopes from 15–50° are assumed to be prone to shallow, planar slides. Terrain prone to rotational slumps was expanded from 20–35° [2] to 15–35° based on observed slumps in Lebanon (e.g [6]). Rockfalls were limited to slopes steeper than 35°. Slopes shallower than 15° were not considered likely to fail in any landslide mode [7,8].

### Multimodal hazard model

Landslide hazard may be quantitatively analyzed through either statistical or physically-based approaches [9]. Physically-based models may be data intensive on regional scales and significantly simplify landslide geometry and processes; however, they provide key advantages over statistical models in quantitative risk assessment. Physically-based models are not dependent on the stasis of present environmental conditions, as are statistical models [54]. Furthermore, because they capture the underlying physical processes which lead to landslide failures, physically-based models are not limited to the area in which there were developed, but are transmissible across geologic, physiographic, and climatic regions. As a key goal of risk assessment is to disaggregate the risk contribution of different triggering scenarios, such as groundwater response during a storm or earthquake shaking, statistical models are less useful for risk assessment when triggering data is not linked to the landslide record. Finally, unlike statistical models, physically-based models do not require detailed landslide inventories, which are rare, costly to develop, often incomplete, and too short to capture large, infrequent events [10,11]. For these reasons, we adopt physically-based models for use in the county of Lebanon, which has significant geologic, physiographic, and climatic diversity; experiences a suite of both short and long return-period landslide-triggering events; and lacks a detailed, national landslide inventory.

We adopt the pixel-based, multimodal coseismic landslide hazard method of Grant et al. [2] and present a parallel set of mode-specific models for rainfall-induced landslides. Simple, physics-based models are used to calculate the factor of safety (FS) against shallow, disrupted slides; rockfall; and rotational, coherent slumps. The model for coseismic rotational slumps was expanded to three dimensions (see formulation of precipitation-induced slump model below). We use the simplified Newmark-sliding block method [12] to convert static FS values into the expected cumulative displacement of a landslide body caused by peak ground accelerations from a probabilistic seismic hazard analysis (PSHA). Failure was anticipated at coseismic displacements of 5 cm, 5 cm, and 15 cm for disrupted slides, rockfall, and coherent failures, respectively, after Grant et al. [2].

The initiation of debris flows was assessed using the shallow landslide model SHALSTAB [13], which couples hydrologic and limit-equilibrium slope stability models to compute the critical daily rainfall to trigger a shallow soil failure (Fig. S1):

(1) $$Q_{cr}=\;\frac{T\text{sin}\beta }{\left(\frac{a}{b}\right)}\left[\frac{c^{\text{'}}}{\gamma_{w}z{cos}^{2}\beta tan\varphi }+\frac{\gamma }{\gamma_{w}}\left(1-\frac{\tan\beta }{\tan\varphi }\right)\right]\;$$

where *Q_cr_* is the critical rainfall [mm/d], *T* is the transmissivity of soil [m^2^/d], *a* is the catchment area [m^2^], *b* is the pixel width [m], *β* is the slope angle [rad], *φ* is the friction angle [rad], *γ_w_* is the unit weight of water [kg/m^3^], *γ* is the unit weight of soil [kg/m^3^], *z* is the soil thickness [m], and *c'* is the effective cohesion including the contribution of vegetation [kN/m^2^]. We assumed a failure plane thickness of *z* = 1 m, common to many shallow landslide studies [[13], [14], [15]]. Adjacent failing cells were combined in a GIS environment to identify the spatial extent and volume of individual debris flow sources. These were classified by volume as low, medium, and high magnitude corresponding to failures of < 800 m^3^, 800–2000 m^3^, and > 2000 m^3^, respectively, after Corominas et al. [16].

Rainfall-induced rockfalls are modeled as simple Culman wedge-like masses [17] including the effects of pore-pressure acting on the failure plane (Fig. S2):

(2) $$FS=\frac{2c\text{sin}\beta }{\gamma h\text{sin}\left(\beta -\alpha \right)\text{sin}\alpha }+\frac{\text{tan}\varphi }{\text{tan}\alpha }-\frac{h_{w}\gamma_{w}\text{sin}\beta \text{tan}\varphi }{2h\gamma \text{sin}\left(\beta -\alpha \right)\text{sin}\alpha }$$

where *FS* is the factor of safety of the rock wedge, *γ* is the unit weight of rock [kN/m^3^], *h* is the height of rock face [m], *β* is the slope angle [rad], *φ* is the friction angle [rad], *γ_w_* is the unit weight of water [kN/m^3^], *h_w_* is the height of water [m], and *c* is the cohesion [kN/m^2^], and *α* is the critical failure plane angle [rad] given by $\alpha =\;\left(\frac{\beta +\;\varphi }{2}\right)$.

To analyze the occurrence of rotational slumps, Grant et al. [2] developed a uniform idealized failure surface that combines larger hillslope conditions with individual pixel properties such as cohesion and friction angle. However, the factor of safety equation was undefined for slopes < 20° and produced extreme failure geometries at the high and low extremes of the susceptible slope ranges. We modify the procedure of Grant et al. [2] by adding a flexible failure depth parameter, *p*, to create realistic failure geometries at slopes of 15–35° in which rotational failures are commonly observed ([[18], [19], [20]]; Fig. S3). To account for wet conditions, the water pore pressure was added to the factor of safety equation, based on a slope-parallel ground water table at a percentage of the maximum failure depth. In addition, we project the radius of failure, *R* [m], perpendicular to the slope to create three-dimensional geometry:

(3) $$FS=\;\frac{2\pi Rbc+(W\text{cos}\alpha -F_{w})\text{tan}\varphi }{W\text{sin}\alpha }$$

where c and φ are the cohesion [kPa] and friction angle [rad] of the soil mass, and α is the basal angle [rad] given by ${\text{α}=\text{sin}}^{-1}\frac{\left(4{(\text{sin}\delta )}^{3}\text{sin}\theta \right)}{3(2\delta -\text{sin}2\delta )}$. The weight of the sliding block, W, is

(4) $$W=\;\frac{\pi \gamma b^{2}}{3}\left(3R-b\right)$$

and the resultant force of pore water pressure, $F_{w}$, is

(5) $$F_{w}=\;\frac{1}{3}\pi \gamma_{w}q^{2}(3R-q)$$

Where *b* and *q* are defined as b=R(1- cosδ ) and q=R(1- cosθ ). The radius of failure is a function of local relief, $R=\frac{H}{4}\left(\frac{\text{cos}\beta }{p{(\text{sin}\beta )}^{2}}+\frac{p}{\text{cos}\beta }\right)$ where *H* is local hillslope relief [m] and *p* is a depth factor empirically fixed at 1.0 to produce the minimum factor of safety in most cases for our study area. The internal angle, δ, is computed as $\delta ={\text{sin}}^{-1}\frac{H}{2R\text{sin}\beta }\;$ [rad] for slopes 15°≤β≤35°, and θ is determined by the level of saturation. The local hillslope relief was determined by a 23 m moving window, selected to capture the major topographical features of our study area.

### Strength parameters

Rock and soil parameters were based on mapped geologic units [21] and adjusted from those estimated by Grant et al. [2] based on field observations of rainfall-induced slope failures (Table S1). Observed coherent failures occurred in a mixture of soil and rock. However, since the coherent rotational model assumes a homogeneous failing mass, single mid-range values of cohesion and friction angle were estimated. Values of friction angle in the debris flow triggering model were increased to 45, 50, and 55° to account for the cohesionless soil assumption in the SHALSTAB model, based on consultation with geotechnical practitioners in Lebanon [13]. Soil transmissivity was coarsely estimated from the hydraulic conductivity in different geologic units (Table S1). The unit weights of soil and rock were assigned to be 20 kN/m^3^ and 23 kN/m^3^ respectively.

The presence and type of vegetation influences the location of shallow precipitation-induced landslides, primarily through its contribution to effective soil cohesion through a root network [22]. Grant et al. [2] observed a clear relationship between shallow coseismic landslide occurrence and the relative normalized difference in vegetation index (NDVI). Root cohesion was accounted for in the initiation of shallow failures based on the NDVI. In moderately and highly vegetated areas (NDVI 0.1 – 0.4 and > 0.4) we adopted conservative values of 3 and 5 kPa respectively, based on the root cohesion contributed by shrubs and trees similar to that in our study area of Lebanon [23].

### Triggering events

Huijer [24] modeled the major seismic sources in Lebanon, producing contour maps of peak ground acceleration (PGA) with a 10% exceedance probability in 50, 100, and 500 years. We adopt model 2a which includes the four major faults as well as a Lebanon region areal source and is the most realistic the five models presented ([24]; Fig. S4).

Rainfall-induced buildup of groundwater pressure is the leading cause of damaging landslides worldwide [25]. Rainfall time-scales significantly control the mode of slope failure. Debris flow and shallow landslide triggering has been observed on short times scales (minute - day) after intense rainfall (e.g [26,27]) whereas deep-seated, rotational slumps are controlled by antecedent rainfall and seasonality [20]. In turn, rainfall-induced shallow landslide triggering can be reasonably predicted using process- or empirically-based models using short term rainfall data [28]. However, hillslope response to antecedent rainfall involves extensive information about soil conditions at depth and complex, time-step infiltration models, making processed based prediction of rainfall-induced deep-seated landslides non-trivial at any scale.

Due to its 15-year civil war (1975–1990), Lebanon lacks long-term pluviometric data [29]. In order to incorporate the geomorphic response to short- and long-term rainfall during major-return period storms while maintaining computational practicality, we combine continuous annual and localized daily rainfall measurements from Lebanon and from this estimate percent saturation of hillslopes. A map of annual average rainfall ([30], Fig. S4) and point data of maximum daily rainfall from eight weather stations within Lebanon were used estimate the hydrologic inputs for each mode of failure. The weather station data was overlaid on the pluviometric zones and was linearly scaled by the average annual rainfall depths, creating a geographically continuous map of scaled “daily” rainfall for 2, 3, 5, 10, 20, 50, and 100-year return-period events (Table S2). As a first order estimation of the percent of hillslope saturation, we adopt a maximum 70% saturation and then linearly scaled saturation levels by the combined short- and long-term precipitation for each return period and pluviometric zone (Table S3).

### Runout

Landslide runout, rather than the initial failure, is the primary source of landslide-related casualties, making the analysis of post-failure motion a key step in landslide risk analyses [31]. We adopt a runout analysis method which combines published empirical relationships, informal inventories in Lebanon, and the experience of Lebanese practitioners appropriate for the large scale and scarce site-specific data of our study area. Under intense rainfall, debris flows can travel long distances in existing topographic channels. We extract flowlines from a 15 m digital elevation model to calculate the runout path from each debris flow source. In the absence of empirical volume – runout data in Lebanon, we limit each path to 750 m, based on an inventory of 69 relict debris flow tracks observed in Google Earth. For each track, we made basic measurements of elevation, source zone slope, runout height and length in a GIS environment. Elevation and slope were poor predictors of runout length (R^2^ of 0.48 and 0.02, respectively); thus, we chose a conservative threshold of 750 m, capturing 80% of the mapped debris flows. While this is likely to overestimate the runout of an isolated debris flow, the densely channeled terrain of the Mount Lebanon and Anti-Lebanon Ranges directs high and low elevation debris flow sources into the same flow path, extending the effective runout zone. Where overlapping, the runout path grids for lower magnitude debris flows were subtracted from the higher magnitudes, since the greatest damage will be done by the highest magnitude debris flow which crosses each pixel. If a pixel is both a debris flow source zone and in the runout path of an upslope debris flow, only the runout was considered in the risk equation for that pixel as the more damaging of the two hazard processes. Coseismic and precipitation-induced rockfall runout zones were calculated using a viewshed analysis from each initiation point. The horizontal extent of the rockfall zone was limited by the maximum and minimum aspect of the source cliff face plus 17 ° based on observed lateral dispersion angles [[32], [33], [34]]. We limit runout based on the “angle of reach” [35] of rockfall observed in Google Earth and during our field campaign in Lebanon, up to a maximum horizontal displacement of 265 m. The reach angles for small (less than 1000 m^3^) and large (greater than 1000 m^3^) rockfall were 42° and 34°, respectively, comparable to global observations [16,36]. The influence of coherent slumping failures was limited to the radius of the failing body, projected as a circular zone in a GIS environment.

### Multimodal hazard output

A landslide hazard map was produced for each mode and triggering scenario (Fig. S5). Our analysis found high debris flow hazard along the steeply-incised river valleys of the Mount Lebanon Range as well as in the northern Anti-Lebanon range where there is little vegetation to inhibit erosion. Coseismic disrupted slides were limited to isolated, sparsely-vegetated zones in the upper reaches of Mount Lebanon’s river valleys and near Hamat. Coseismic and rainfall-induced rockfall shared similar extents, primarily limited to the exposed cliff bands in the Sannine and Keserouane formations of Western Lebanon. Extensive coseismic rockfall is also predicted in the steep valleys of the southern Litani River near the Yammouneh fault. Coherent failures are infrequent and concentrated in Mount Lebanon’s weak sandstone. We undertook two field campaigns 2015–2017 to qualitatively assess the results of the multimodal hazard model. These included both a broad reconnaissance across the region as well as detailed assessments at 59 sites. In general, the model correctly identified terrain susceptible to different modes of failure (Fig. S6). However, few of the landslide deposits observed in the field were associated with a triggering event, so we could not assess the frequency component of the multimodal hazard model. We do not attempt to quantify the uncertainty in the hazard output, as statistical descriptions of the variation in geotechnical strength parameters, rainfall accumulations, effective root cohesion, etc. were not available.

### Elements at risk

Although conceptually simple, mapping elements at risk on a country-scale presents a practical challenge in the amount and detail of information which must be collected in the ever-developing human-built environment. Due to the high resource demands of collecting this data on a regional scale, open, accurate, and high-resolution population and infrastructure data is scarce [37]. Recent advances in remote sensing have expanded the range of high resolution, satellite-derived data (e.g. landcover) with immediate benefit to the fields of urban planning, humanitarian aid, global development, etc. [[38], [39], [40]]. Human presence for risk analyses is evaluated through imagery-based mapping of human infrastructure, typically building stock, inventories of building location and footprint, or built-up presence, a binary map of made-made surfaces identified by textural features rather than spectral signature [41,42]. In disaster risk management, building stock data has the advantage of identifying individual structures at risk and associating them with unique attributes such as material, height, physical vulnerability, or number of residents. However, developing such inventories is costly and time-consuming, infeasible at greater than city scale [43]. Although built-up area does not allow for delineation of individual elements at risk, it does provide the location, extent, and density of human settlement in an automated, and consistent fashion, which makes it practical tool for risk analyses when large areas or rapid assessments are needed, such as in crisis management or tracking transient displaced peoples [40,43]. We utilize binary grids produced by the Global Human Settlement Layer which assess the build-up presence (1 = yes, 0 = no) at 38 m pixel resolution using Landsat Imagery collected from 2013 to 2014 ([44]; Fig. S7). Rather than identifying individual buildings at risk, we adopt the built-up grid as areas throughout which human activity is distributed. As only one epoch of built up area is available during the span of the Syrian crisis, we are unable to assess the impacts of the refugee influx on infrastructure expansion in Lebanon. We resampled built-up grids to 15 m and subtracted the Lebanese road network gridded from Open Street Map data (©Open Street Map, accessed 25 July 2017) to produce separate grids of residential area and road network. In this work, we do not differentiate between commercial and residential area.

The UNHCR has documented the Syrian refugee population in Lebanon since the outset of the Syrian crisis in 2011. In support of their aid distribution activities, the UNHCR published a map series showing the registered Syrian refugee population by cadastre on a bimonthly basis from 2013 to 2016 with infrequent updates 2017–2018. We digitized this map series on three-month intervals to assess the evolving refugee population in Lebanon from 2013 to 2018. On the UNHCR maps, population is given both as discrete numerical values and on a binned color scale. Where numerical values are absent for individual cadastres, the minimum population indicated by the background color was used, resulting in an underestimation of the national registered Syrian refugee population of about 5%. This unaccounted-for minority of registered Syrian refugees adds to the approximately 500,000 unregistered Syrian refugee population for which there is no spatial distribution data, representing a significant, unavoidable source of non-conservativism in our risk analysis, especially since the forces which dissuade refugees from registering with the UNHCR also promote marginalization and consequently higher vulnerability [45,46].

In the absence of a national census since 1932, the United Nations Statistics Division’s Population Prospects has been the most accurate source of population data in Lebanon. Prior to the outbreak of the Syrian crisis, Lebanon’s population was predicted to increase by ˜150,000 from 2013 to 2018 [47]. To support their work identifying vulnerable Lebanese, the UNHCR used the UN population projections to estimate the distribution of the native Lebanese population by cadastre in 2015. We adopt these values as the static, native Lebanese population from 2013 to 2018, acknowledging that this is an approximation. Since most cadastres are small (˜6 km^2^), containing a single urban cluster, we assume a constant population density within each cadastre, uniformly distributing the number of Lebanese and Urban Syrian refugees among the built-up pixels, resulting in 15 m gridded populations.

Built-up area maps of Lebanon have not been produced at sufficient temporal resolution to track the transience of informal tented settlements (ITS) in Lebanon or spatial resolution to resolve small settlements. However, the UNHCR and its partners have performed periodic, country-wide inventories to identify the coordinate location, number of tents, and population of ITS in Lebanon since 2013 in order to provide targeted humanitarian aid. We have access to ten ITS inventories from February 2014 – December 2017. To convert point coordinates to areal settlement extents, we apply a buffer of 10 m to every settlement of ten or fewer tents, increasing the buffer by 1 m for every tent in a settlement beyond ten. This guarantees that every settlement will be represented by at least one 15 m pixel and most or all of the settlement will be enclosed within the buffered zone. ITS zones were then rasterized at 15 m resolution (Figure S7b). The density and layout of informal settlements varies widely across Lebanon, influencing the accuracy of our ITS buffering method. Dense, elongate camps are more likely to be surrounded by a wide buffer of unoccupied area zoned as encamped, while sparse, blocky camps are more likely to have internal areas not occupied by tents but still within an ITS zone. To minimize inflation of risk by over-zoning encamped areas, we calculate human exposure on a per-camp basis. Since unoccupied areas within and around camps are included, the byproduct is an implicit assumption that encamped refugees spend significant time outdoors in the vicinity of the ITS.

### Exposure

The human exposure within a buffered settlement is the area occupied by individuals divided by the total area of the settlement. Limited data is available on the shelter density of refugees in Lebanon, but the UNHCR estimates that 53% of households in ITS are overcrowded at less than 4.5 m^2^ per person based on systematic interviews [48]. Thus, the exposure within an ITS is:

(6) $$E_{\left(ITS\right)}=\;\frac{4.5P}{A_{\left(ITS\right)}}$$

where *P*, is the population of the settlement and *A_(ITS)_* is the area of the buffered settlement [m]. The exposure of urban populations was estimated based on average building size and the expected zone of influence for each hazard mode (Table S4). We assumed an average building height of 3 floors based on a review of 164 for-sale residences across Lebanon as of 20 Dec. 2017 [49]. Source zones of shallow failures were assumed to affect the entire bottom floor of a building through undercutting and scour. As channelized failures, debris flows were assumed to affect only 20% of the lowest floor of a building. Due to their size and depth, coherent failures affect the entire building. Fragmentation and spread of rockfall debris were not accounted for in the runout zonation; therefore we assume that only 10% the built-up pixels in a runout zone will actually be impacted by significant debris, and that, due to bouncing, debris will affect 20% of the lowest two floors of a building. Human occupants are assumed to spend equal time in all parts of a home, resulting in exposure values less than one.

### Vulnerability

At a minimum, vulnerability is a function of the type of hazard, its magnitude, and physical characteristics of the element at risk. For buildings this may include construction material, orientation, number of windows, height, etc. For humans, vulnerability includes a host of predisposing factors to physical injury, such age, health, gender, and mobility, as well as less intuitive intangibles: economic station, language, food security, educational level, etc. For this reason, virtually all assessments of human vulnerability to landslides have been subjective, based on expert judgement and a limited set of fatal landslide inventories [50]. Data on landslide fatalities in Lebanon is scarce and does not include qualitative information on landslide magnitude, preventing the estimation of region-specific human vulnerability values (Table S5). Therefore, we follow existing literature to estimate the physical vulnerability of the urban (Lebanese and Syrian) and encamped populations in Lebanon (Table S4). Vulnerability of urban Lebanese and Syrians was assessed as that of indoor populations based on fatal landslide incidents in Hong Kong and Australia [50,51]. Due to the temporary materials and irregular construction of ITS, encamped residents are assessed vulnerability values consistent with an outdoor population, although we note that the quality and protection of ITSs varies widely [52]. Vulnerability to debris flow runout was assessed at an order of magnitude higher than that of hillslope source zones, due to greater channelization and velocity, after Michael-Leiba et al. [51]. Coherent failures generally develop slowly allowing residents to evacuate; thus, a nominal human vulnerability of 0.0001 was adopted.

### Conclusion

In this work, we present a flexible and efficient method for quantitative landslide risk analysis and apply it to the country of Lebanon. We propose a suite of mode-specific, physically-based failure models for coseismic and rainfall-induced landslides. Although physically-based models simplify landslide geometry and processes, our method significantly expands traditional infinite-slope-only hazard analyses and provides a modular framework into which refined mode-specific models can be added. Further limitations of the multimodal method are discussed in Pollock et al. [53]. We perform a deterministic landslide hazard analysis of the country of Lebanon, commensurate with the level of input data presently available for the country. Exposure values inferred from humanitarian and real estate data and published vulnerability estimates were combined with the landslide hazard to produce maps of landslide risk for the country of Lebanon (see [53]).
