# Supplementary material for: Multimodal method for landslide risk analysis
Source: MethodsX. 2019 Apr 16;6:827–36. doi: 10.1016/j.mex.2019.04.012 (PMC6484206; doi:10.1016/j.mex.2019.04.012)
Supplement: Supplementary file 1 [file mmc1.docx]

**Tables and Figures**

**Table S1.**  Geologic units and geotechnical parameters used in this study. Geologic mapping after Dubertret (1945).

| Age | Formation | Geologic Unit | Rock-slope failures | | Coherent rotational slumps | | Disrupted soil slides | |
| --- | --- | --- | --- | --- | --- | --- | --- | --- |
|  |  |  | Cohesion | Phi | Cohesion | Phi | Cohesion | Phi |
| Quaternary | Quaternary Heterogeneous Deposits | qd, qm | 20 | 20 | 20 | 20 | 20 | 20 |
|  |  | ql, qs, qta, qaoi | 20 | 30 | 20 | 30 | 20 | 30 |
|  |  | q, qcg, ql1 | 20 | 25 | 20 | 25 | 20 | 25 |
|  |  | qar | 20 | 18 | 20 | 18 | 20 | 18 |
|  |  | a, ad | 15 | 33 | 15 | 33 | 15 | 33 |
|  |  | Alluvium | 15 | 33 | 15 | 33 | 15 | 33 |
|  |  | gravel slopes | 15 | 33 | 15 | 33 | 15 | 33 |
|  |  | Alluvial fans | 15 | 33 | 15 | 33 | 15 | 33 |
|  |  | Silt and mud | 15 | 33 | 15 | 33 | 15 | 33 |
|  |  | Rocky | 25 | 33 | 25 | 33 | 25 | 33 |
|  |  | Fill | 15 | 33 | 15 | 33 | 15 | 33 |
|  |  | Scree | 25 | 33 | 25 | 33 | 25 | 33 |
|  | Quaternary Basalt | qb | 20 | 20 | 20 | 20 | 26 | 24 |
| Neogene | Misc. Miocene units | m | 48 | 21 | 48 | 21 | 32 | 25 |
|  |  | m2, m2a, m2b | 55 | 25 | 55 | 25 | 35 | 22 |
|  |  | mcg, ml, ml1 | 48 | 21 | 48 | 21 | 14 | 26 |
|  | Misc. Pliocene Units | p | 57 | 25 | 57 | 25 | 67 | 22 |
|  |  | p1 | 62 | 32 | 62 | 32 | 57 | 26 |
|  | Misc. Neogene Units | n2, n | 58 | 30 | 58 | 30 | 62 | 25 |
|  |  | ncg | 62 | 32 | 62 | 32 | 57 | 26 |
|  | Neogene Basalt | bp, bp1-2, bp3 | 62 | 27 | 62 | 27 | 27 | 30 |
|  |  | bn | 52 | 24 | 52 | 24 | 26 | 24 |
| Paleogene | Numilitic Eocene Formation | e2, e2a | 42 | 18 | 44 | 21 | 18 | 21 |
|  |  | e2b | 44 | 20 | 44 | 21 | 24 | 20 |
| Cretaceous | Chouf Sandstone | c1, c1-2, c1-2a, c2 | 48 | 21 | 33 | 21 | 18 | 21 |
|  | Abieh Formation | c2a | 45 | 20 | 45 | 20 | 47 | 20 |
|  | Mdairej Formation | c2b | 68 | 33 | 77 | 34 | 35 | 25 |
|  | Hammana Formation | c3, c3b, c3-4 | 48 | 21 | 48 | 21 | 17 | 21 |
|  | Sannine Formation | c4, c4-5 | 59 | 24 | 45 | 22 | 34 | 18 |
|  | Maameltain Formation | c5 | 59 | 24 | 59 | 24 | 35 | 25 |
|  | Chekka Formation | c6 | 48 | 21 | 33 | 20 | 18 | 21 |
|  | Cretaceous Basalt | bc, bc1, bc2a, bc2b, Bc | 56 | 27 | 56 | 27 | 13 | 24 |
| Jurassic | Chouane Formation | j1-3, j2, j3, j | 61 | 26 | 61 | 26 | 53 | 28 |
|  | Keserouane Formation | j4, j6 | 61 | 26 | 61 | 26 | 35 | 20 |
|  | Bikkfaya Formation | j6a | 84 | 38 | 84 | 38 | 113 | 34 |
|  | Salima Formation | j7 | 45 | 21 | 45 | 21 | 35 | 20 |
|  | Jurassic Basalt | bj, bj6, bj5 | 52 | 24 | 18 | 24 | 13 | 24 |

**Table S2.** Scaled daily rainfall in each pluviometric zone.

| Mean Annual Rainfall (mm/yr) | Return Period (yr) | | | | | | |
| --- | --- | --- | --- | --- | --- | --- | --- |
|  | 2 | 3 | 5 | 10 | 20 | 50 | 100 |
|  | Scaled Daily Rainfall (mm/day) | | | | | | |
| 100 | 60 | 69 | 78 | 89 | 98 | 108 | 114 |
| 200 | 60 | 69 | 79 | 92 | 104 | 120 | 133 |
| 300 | 59 | 69 | 80 | 94 | 110 | 132 | 152 |
| 400 | 58 | 68 | 80 | 97 | 116 | 145 | 171 |
| 500 | 57 | 68 | 81 | 100 | 122 | 157 | 190 |
| 600 | 56 | 67 | 82 | 103 | 128 | 169 | 209 |
| 700 | 55 | 67 | 82 | 106 | 134 | 182 | 228 |
| 800 | 54 | 67 | 83 | 109 | 140 | 194 | 247 |
| 900 | 53 | 66 | 83 | 111 | 147 | 206 | 266 |
| 1000 | 52 | 66 | 84 | 114 | 153 | 219 | 285 |
| 1100 | 51 | 65 | 85 | 117 | 159 | 231 | 304 |
| 1200 | 50 | 65 | 85 | 120 | 165 | 243 | 323 |
| 1300 | 49 | 65 | 86 | 123 | 171 | 256 | 342 |
| 1400 | 48 | 64 | 87 | 126 | 177 | 268 | 361 |

**Table S3**. Hillslope saturation in each pluviometric zone.

| Mean Annual Rainfall (mm/yr) | Return Period (yr) | | | | | | |
| --- | --- | --- | --- | --- | --- | --- | --- |
|  | 2 | 3 | 5 | 10 | 20 | 50 | 100 |
|  | Saturation (%) | | | | | | |
| 100 | 12 | 13 | 15 | 17 | 19 | 21 | 22 |
| 200 | 12 | 13 | 15 | 18 | 20 | 23 | 26 |
| 300 | 11 | 13 | 15 | 18 | 21 | 26 | 29 |
| 400 | 11 | 13 | 16 | 19 | 22 | 28 | 33 |
| 500 | 11 | 13 | 16 | 19 | 24 | 30 | 37 |
| 600 | 11 | 13 | 16 | 20 | 25 | 33 | 41 |
| 700 | 11 | 13 | 16 | 21 | 26 | 35 | 44 |
| 800 | 10 | 13 | 16 | 21 | 27 | 38 | 48 |
| 900 | 10 | 13 | 16 | 22 | 28 | 40 | 52 |
| 1000 | 10 | 13 | 16 | 22 | 30 | 42 | 55 |
| 1100 | 10 | 13 | 16 | 23 | 31 | 45 | 59 |
| 1200 | 10 | 13 | 17 | 23 | 32 | 47 | 63 |
| 1300 | 9 | 13 | 17 | 24 | 33 | 50 | 66 |
| 1400 | 9 | 12 | 17 | 24 | 34 | 52 | 70 |

**Table S4.** Exposure and vulnerability estimates based on average building size and literature.

| **Hazard** | **Magnitude** | **Zone** | **Exposure**  (urban) | **Vulnerability** (urban) | **Vulnerability**  (encamped) |
| --- | --- | --- | --- | --- | --- |
| Debris Flow/  Disrupted | High | Runout | 0.067 | 0.050 | 1.00 |
|  |  | Source | 0.333 | 0.005 | 0.10 |
|  | Medium | Runout | 0.067 | 0.025 | 0.30 |
|  |  | Source | 0.333 | 0.0025 | 0.03 |
|  | Low | Runout | 0.067 | 0.010 | 0.10 |
|  |  | Source | 0.333 | 0.001 | 0.01 |
| Coherent |  |  | 1.000 | 0.0001 | 0.0001 |
| Rockfall |  |  | 0.013 | 0.050 | 0.50 |

**Table S5**. Inventory of fatal and significant non-fatal landslides in Lebanon.

| *Fatal Landslide Events in Lebanon 1975 – 2015* | | | | | |
| --- | --- | --- | --- | --- | --- |
| Date | Event | Location | Deaths | Description | Source |
| Feb. 1975 | landslide | Faraiya, Mount Lebanon | 5 | Damaged electric and communication network; $5M in losses | Abdallah (2007) |
| 12 Mar. 1983 | avalanche | Bcharre, North | 1 | Destroyed orchard | www.desinventar.net |
| Dec. 1983 | landslide | Bekaa | 20 |  | www.emdat.be |
| 1984 | landslides / avalanches | North, Mount Lebanon, Baalbek-Hermel | 90 | Extensive damage to infrastructure; $68 M in losses | Abdallah (2007) |
| 09 Feb. 1992 | landslide | Beirut | 1 | Building collapsed, 2 injured | www.desinventar.net |
| 18 Mar. 1992 | landslide | Hazerta, Bekaa | 20 | Several houses buried | Abdallah (2010) |
| 07 Feb. 1997 | landslide | Mansourieh, Mount Lebanon | 2 | One house destroyed | www.desinventar.net |
| 09 Oct. 2004 | landslides | Ehden, North | 2 | 6 injured | www.desinventar.net |
| 07 Feb. 2005 | avalanche / rock fall | Bcharre, North | 1 | Deceased was a Syrian worker; house destroyed | www.desinventar.net |
| 05 Jan. 2013 | landslide | Beirut | 1 | A child died when he and his mother were carried away by a bluff collapse | english.al-akhbar.com/node/14602 |
| 18 Sept. 2013 | landslide | Karak, Bekaa | 1 | Deceased was a Syrian worker; 4 injured; landslide in a construction site | www.nna-leb.gov.lb/en/show-news/14119/Landslide-kills-one-Syrian-injures-four-in-Zahle |
| 13 Mar. 2014 | rock fall | Roumieh, Mount Lebanon | 1 | 3 injured; rock fall into a construction site | data.nasa.gov/Earth-Science/Global-Landslide-Catalog/h9d8-neg4/data |
| 27 Mar. 2014 | landslide | Kfar Tebnit, Nabatiyeh | 1 | Deceased was a Syrian worker; 6 injured | www.lbcgroup.tv/news/d/lebanon-news/147134/one-killed-6-others-injured-in-kfartibnit-landslid/en |
| *Other significant events* | | | | | |
| 09 July 551 | rotational slumps | Chekka, Mount Lebanon |  | Triggered by M 7.5 Beirut-Tripoli earthquake | Abdallah (2007) |
| 20 May 1202 | rock falls | Mount Lebanon | 200 | Triggered by M 7.5 earthquake in the Bekaa Valley | Daeron et al. (2005) |
| 18 Jan. 1767, 30 Nov. 2015 | landslide / rock fall | Kfar Nabrakh, Mount Lebanon |  | 1767: numerous deaths, major property damage  2015: 17 houses evacuated | El Mohtar et al. (2016) |
| 1860-1983 | many | Hammana, Mount Lebanon |  | Perennially unstable region; failures triggered by earthquakes in 1924 and 1956 | Khawlie and Hassanain (1984) |
| 16 Feb. 2000 | landslide | Biaqout, Mount Lebanon |  | Destroyed three apartment buildings; 300 people evacuated | www.dailystar.com.lb/News/Lebanon-News/2000/Feb-17/27252-landslide-causes-buildings-to-collapse-after-rain-soaks-foundations.ashx |
| 25 Mar. 2000 | landslide | Aayoun Orghosh, Mount Lebanon |  | Destroyed 10 houses and several ski resorts | Abdallah (2010) |
| 26 Mar. 2003 | landslide | Cheikh Taba, Akkar |  | Destroyed an apartment building; $300,000 in losses | www.desinventar.net |


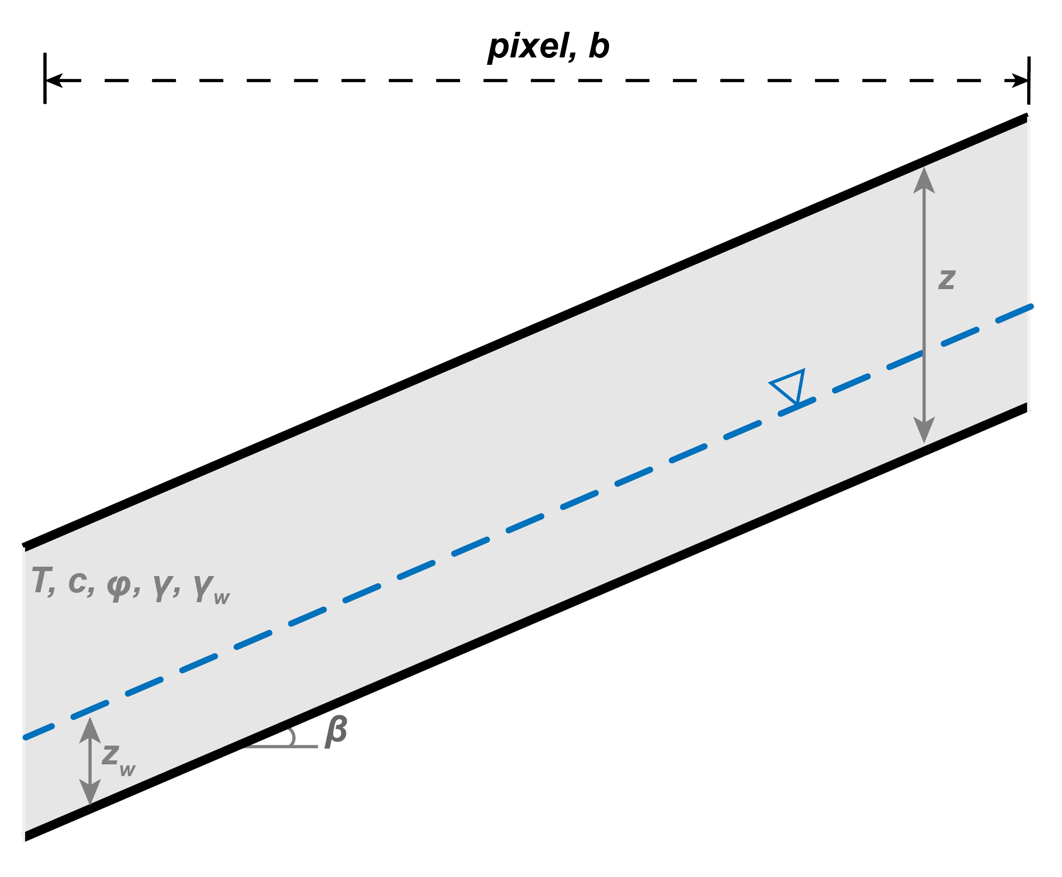


**Figure S1.** Geometry of debris flow initiation model.


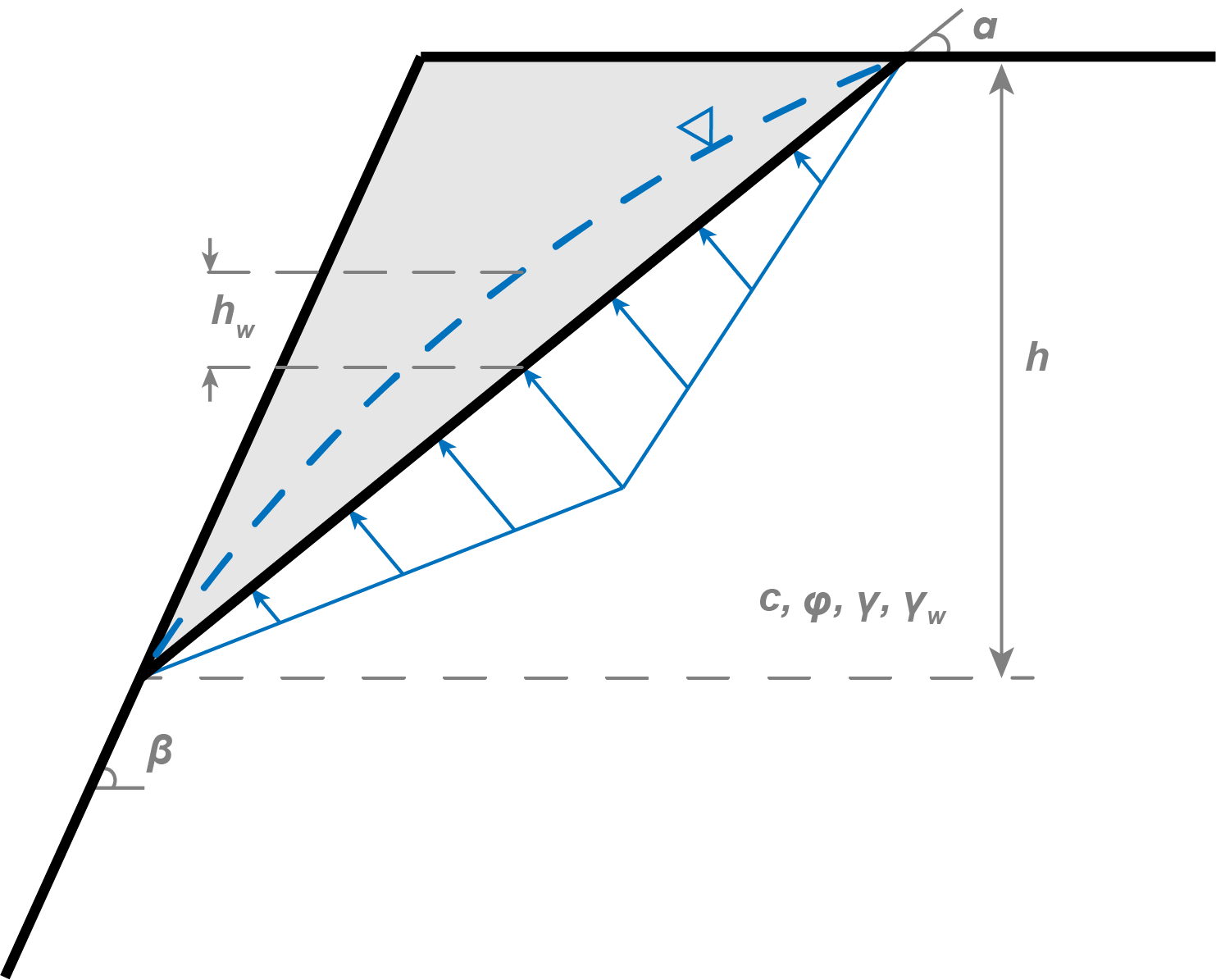


**Figure S2.** Geometry of precipitation-induced rockfall model.


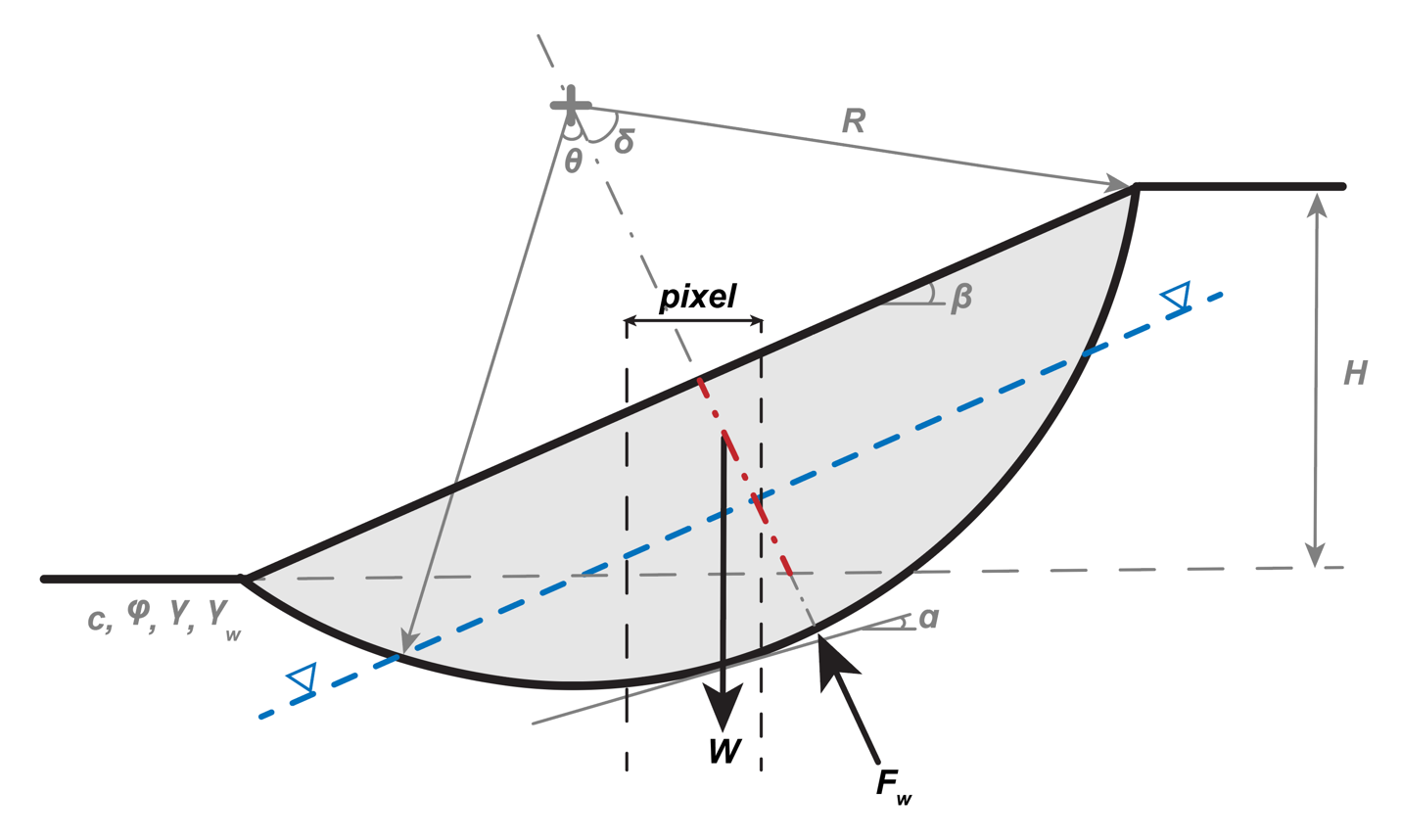

**Figure S3.** Geometry of rotational slump model. The depth of failure is determined as a percentage, *p*, of the perpendicular hillslope bisector segment in red. *p* may be greater than 100% (as shown).


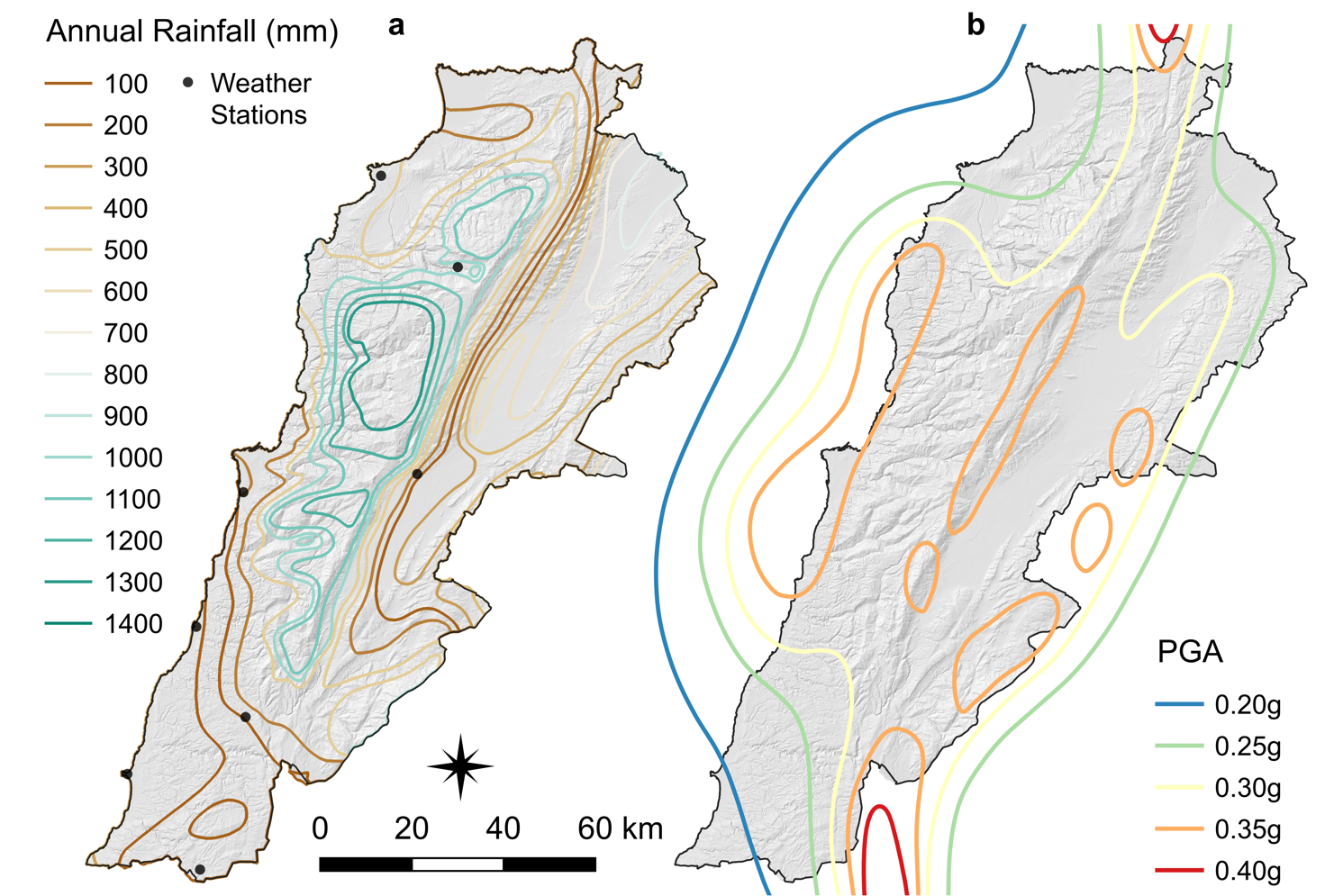


**Figure S4.** Multimodal landslide triggers. (a) Annual rainfall (Plassard, 1971) and (b) 949-year (10% in 100 years) return period PSHA ground shaking intensity contours (Huijer, 2010).

**
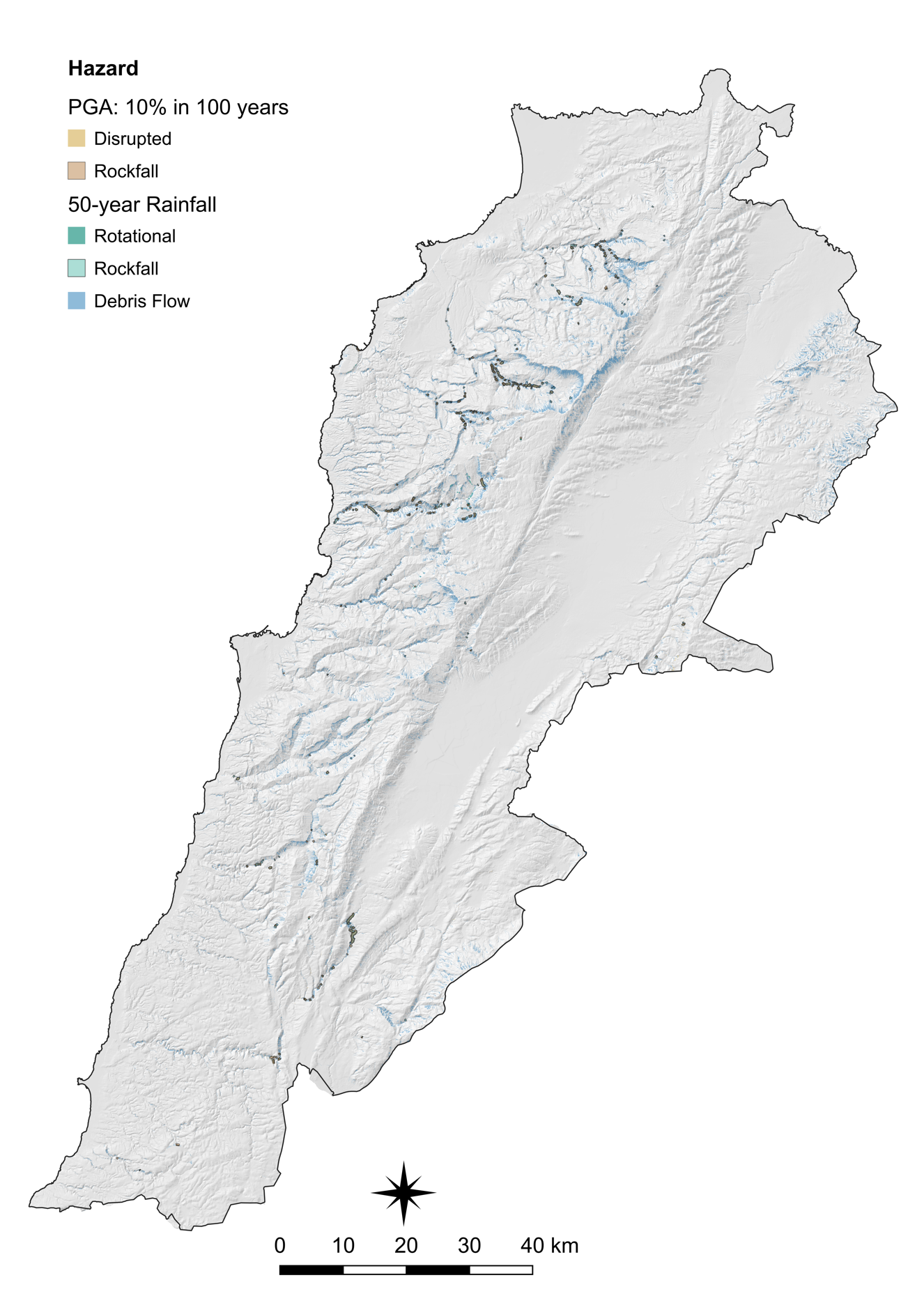
**

**Figure S5:** Multimodal landslide hazard output for 949-year (10% in 100 year) return period PSHA ground shaking intensities and for a 50-year rainfall.

**
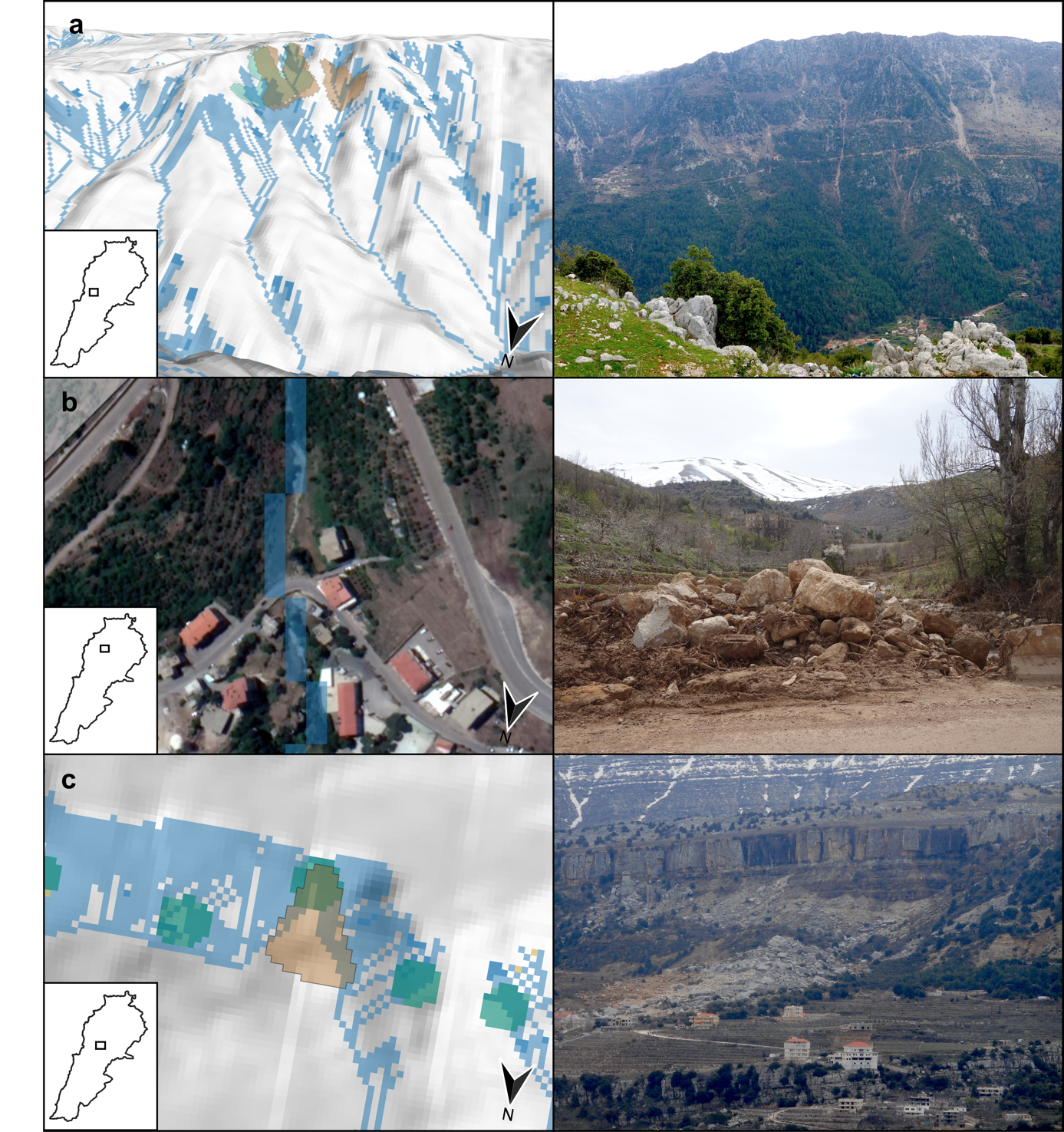
**

**Figure S6:** Multimodal hazard model field observations. (a) Debris flow channels and a rockfall source zone above the towns of Aabra and Chouaneh, (b) A recent debris flow deposit in Hasroun, (c) A large rockfall above Ghabat. Rotational slumps were observed in the vicinity. Base images from Google Earth^TM^.


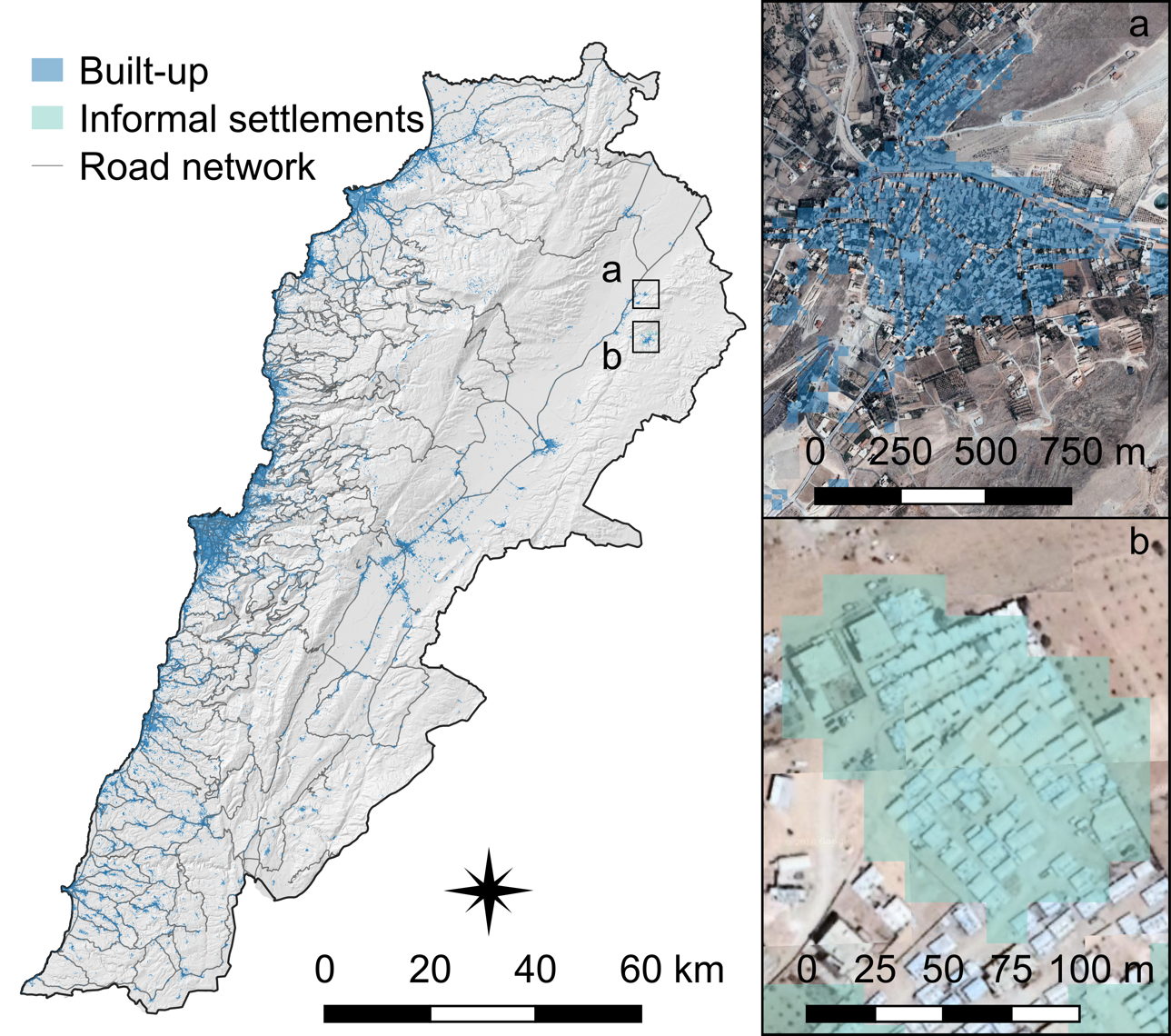


**Figure S7.** Elements at risk. (a) Built-up area in the city of Ras Baalbek. (b) Informal tented settlements on the outskirts of Arsal. Base images from Google Earth^TM^.

**References:**

Abdallah, C. (2007). *Assessment of remote sensing and geographic information systems for the study of mass movements in Lebanon* (Doctoral dissertation). Paris: University of Pierre and Marie Curie

Abdallah, C. (2010). Spatial distribution of block falls using volumetric GIS-decision-tree models. *International Journal of Applied Earth Observation and Geoinformation*, 12(5), 393-403. <https://doi.org/10.1016/j.jag.2010.05.008>

Daëron, M., Klinger, Y., Tapponnier, P., Elias, A., Jacques, E., Sursock, A. (2005). Sources of the large A.D. 1202 and 1759 Near East earthquakes. *Geology*, 33(7), 529-532. <https://doi.org/10.1130/G21352.1>

Dubertret, L. (1945). “Cartes geologiques a l’echelle de 1/50 000 (Hamidieh, Halba, Batroun, Tripoli, Sir El Dannieh, Jbail, Qartaba, Baalbeck, Beirut, Zahle, Rayak, Saida, Jezzine, Rachaya). Republique Libanaise, Ministere des Travaux Publics, Beirut

El Mohtar, C., Abou-Jaoude, G., Abdallah, C., Harb, J. (2016). The Kfarnabrakh landslide of November 30th, 2015 (Geotechnical Extreme Events Reconnaissance-047). Arlington: National Science Foundation

Huijer, C. (2010). Implications of the recent mapping of the offshore thrust fault system on the seismic hazard of Lebanon. M.S. thesis, American Univ. Beirut, Beirut

Khawlie, M., Hassanain, H. (1984). Failure phenomena and environmental control of the relatively unstable Hammana area, Lebanon. *Eng. Geol.*, 20(3), 253-264, <https://doi.org/10.1016/0013-7952(84)90005-X>

Plassard, J. (1971). Pluviometric map of Lebanon at a scale of 1:200,000. Govt. of Lebanon, Ministry of Public Works and Transport, Beirut
